# TACC3 Is Important for Correct Progression of Meiosis in Bovine Oocytes

**DOI:** 10.1371/journal.pone.0132591

**Published:** 2015-07-13

**Authors:** Mahdi Mahdipour, Ana Rita Canhoto Leitoguinho, Ricardo A. Zacarias Silva, Helena T. A. van Tol, Tom A. E. Stout, Gabriela Rodrigues, Bernard A. J. Roelen

**Affiliations:** 1 Department of Farm Animal Health, Faculty of Veterinary Medicine, Utrecht University, Yalelaan 104, 3584 CM, Utrecht, The Netherlands; 2 Centro de Biologia Ambiental e Departamento de Biologia Animal, Faculdade de Ciências, Universidade de Lisboa, 1749–016, Lisboa, Portugal; 3 Department of Equine Sciences, Faculty of Veterinary Medicine, Utrecht University, Yalelaan 104, 3584 CM, Utrecht, The Netherlands; Inner Mongolia University, CHINA

## Abstract

Transforming acidic coiled-coil (TACC) proteins are key players during mitosis via stabilization of the spindle. The roles of TACCs during meiosis are however less clear. We used bovine oocytes to study the expression and function of TACC3 during meiosis. *TACC3* mRNA was detected in bovine oocytes during meiosis using qRT-PCR, and while it was also expressed in cleavage stage embryos, its expression was down-regulated at the morula and blastocyst stages. Immunofluorescence was used to demonstrate that TACC3 co-localized with tubulin in the metaphase I and II spindles. However, TACC3 was not detected at anaphase or telophase of the first meiotic division. Aurora A, which is known to phosphorylate and activate TACC3 in mitotic cells, showed a similar pattern of gene expression to that of *TACC3* in meiotic oocytes and preimplantation embryos. Aurora A protein was however only very transiently associated to the meiotic spindle. Pharmaceutical inhibition of Aurora A activity inhibited TACC3 phosphorylation but did not prevent TACC3 appearance in the spindle. Inhibiting Aurora A activity did however lead to abnormal meiotic spindle formation and impaired maturation of bovine oocytes. Similar results were obtained by knock-down of *TACC3* expression using siRNA injection. These results suggest that TACC3 is important for stabilizing the meiotic spindle, but phosphorylation of TACC3 by Aurora A is not required for its recruitment to the meiotic spindle although phosphorylation of TACC3 by other kinases cannot be excluded.

## Introduction

During its preparation for fertilization, an oocyte reduces its DNA content to a haploid set of chromosomes via a process known as meiosis. Abnormal segregation of chromosomes can lead to numerical chromosome abnormalities, i.e. aneuploidy, which is generally incompatible with normal embryonic development. Whereas in fetal and adult tissues, abnormally dividing (mitotic) cells can be eliminated by for example apoptosis, abnormal chromosome segregation in oocytes would lead to generalized embryonic or fetal aneuploidy, which is recognized as a major cause of pregnancy loss [1].

Within ovarian follicles, immature mammalian oocytes are arrested at prophase I of the first meiotic division and remain in this state until the follicle is recruited for development and oocyte maturation is triggered. The oocyte is enclosed by somatic cells known as cumulus cells and together with other somatic cells in the theca layers they are organized into ovarian follicles. These follicles go through several phases of growth and development that prepare the oocyte for ovulation, fertilization and embryo formation. During early follicle development the oocyte remains arrested in meiosis at the so-called germinal vesicle (GV) stage [2]. In response to a steep pre-ovulatory rise in the circulating luteinizing hormone (LH) concentrations, germinal vesicle breakdown (GVBD) is triggered and meiosis resumes. Following formation of the first meiotic spindle (MI) and segregation of the homologous chromosomes, an asymmetric cell division results and the first polar body is extruded. Exactly how this asymmetry is orchestrated is not known. The oocyte subsequently rearrests at the metaphase stage of the second meiotic division (MII); this is the stage at which the oocytes of most mammalian species are ovulated. The oocyte is now ready to be fertilized and support embryo development. Indeed the oocyte’s cytoplasm is critical for ensuring correct reprogramming of the male pronucleus after fertilization and erasing and replacing epigenetic marks in the embryonic DNA [3,4]

Proper chromosomal alignment and segregation requires correct formation of the meiotic spindles. The spindle is a complex structure composed of microtubules (MTs) that aggregate at the poles of the spindle in the microtubule organizing centres (MTOCs), and attach to the chromosomes via kinetochores [5,6]. The spindle enables the genetic material to be organized and eventually segregated. Correct regulation of this segregation process is vital during both mitosis and meiosis. Various proteins including transforming acidic coiled-coil (TACC) containing proteins [7,8], Aurora kinases [9,10] and TPX2 [11,12] are involved in microtubule dynamics and are important for correct spindle assembly.

In mammals, three TACC proteins are expressed from 3 different genes: TACC1, TACC2 (also known as AZU-1 and ECTACC) and TACC3 (also known as AINT and ERIC1) [13]. During mitosis, the TACC proteins localize around the centrosomes and are important for the organization of the MTOCs [7,14]. TACC3 in particular is thought to increase the stability of centrosomal microtubules during mitosis [7]. TPX2 is another microtubule associated protein that is important for the progression of meiosis [11,12]. Aurora kinases have been reported to play a role in cell-cycle regulation, chromosomal segregation, maturation and cytokinesis [15,16]. Importantly, Aurora A is able to phosphorylate TACC3 and, in so doing, recruit it to and stabilize it on the microtubules, thereby helping to ensure proper cell division [7–9]. Indeed phosphorylation by Aurora A is required for TACC3 to execute its microtubule stabilizing function [9]. In mouse oocytes, it has been demonstrated that Aurora A can, in turn, be activated by TPX2 [11]. The importance of TACC3 has been demonstrated in mice by genetic deletion, which results in embryonic death during mid- to late gestation [17]. As a result of the embryonic lethality, the function of TACC3 during oocyte meiosis remains unclear. Compared to mitosis, little is known about the mechanisms that drive and control meiosis in oocytes.

Here, we show that TACC3 is expressed around the metaphase spindle during meiosis I and II in bovine oocytes. Direct knock-down of *TACC3* expression and indirect inhibition by targeting Aurora-A kinase led to aberrant spindle formation. The results suggest a function for TACC3 in maintaining meiotic spindle stability.

## Materials and Methods

### Oocyte collection, maturation and fertilization

Bovine ovaries were collected from a slaughterhouse (J. Gosschalk en Zn. B.V. Epe, the Netherlands) and transported at room temperature in a polystyrene box, arriving at the laboratory within 2 h after slaughter. After washing and removal of extraneous connective tissue, the ovaries were transferred to a flask containing 0.9% NaCl supplemented with penicillin/streptomycin and maintained at 30°C in a water bath. Cumulus oocyte complexes (COCs) were recovered by aspiration from 2–8 mm follicles and selected for maturation and fertilization based on the presence of a multi-layered cumulus complex, as described previously [18]. In short, oocytes were cultured in maturation medium and fertilized after 22–24 h of culture by co-incubation with 0.5 x 10^6^ /ml frozen-thawed sperm from a bull of proven fertility. Zygotes were denuded of their cumulus investment by vortexing and transferred to synthetic oviductal fluid (SOF) 18–20 h post-fertilization. At day 5 of culture, cleaved embryos were transferred to fresh SOF and cultured until day 8 in a humidified incubator at 39°C, 5% CO_2_ and 7% O_2_.

### RNA isolation and cDNA synthesis

Oocytes and embryos were rinsed in PBS and stored at -80°C until RNA extraction. Isolation of total RNA was performed using an RNeasy Micro Kit (Qiagen, Valencia, CA, USA) as per the manufacturer’s instructions. In short, each sample was lysed in 150 μl of lysis buffer, mixed (1:1) with 70% ethanol and pipetted directly onto an RNA-binding column. After DNAse treatment, the column was washed, followed by elution with 18 μl RNAse-free water. Reverse transcription (RT) was performed in a total volume of 20 μl made up of 10 μl of sample RNA, 4 μl of 5 x RT buffer (Invitrogen, Breda, The Netherlands), 8 units RNAsin (Promega, Leiden, The Netherlands), 150 units Superscript III reverse transcriptase (Invitrogen), 1.8 units per ml of random primers (Invitrogen) and containing final concentrations of 10 mM DTT (Invitrogen) and 0.5 mM dNTP (Promega). The mixture was incubated for 1 h at 55°C, followed by 5 min at 80°C before storage at –20°C. Minus RT blanks were prepared from 5 μl of the same RNA sample under the same conditions, but without addition of reverse transcriptase.

### PCR primer design

Primer pairs used for RT-PCR were designed using the bovine coding sequences and software available from the National Center for Biotechnology Information (http://www.ncbi.nlm.nih.gov); preferably, each primer of a pair was located on a separate gene exon (Table 1). All PCR products were sequenced (ABI Genetic analyzer, Applied Biosystems, Foster City, CA, USA) to verify specificity.

**Table 1:** List of primers used for RT-PCR. L and R refer to left and right primer. The start and end positions of the primers correspond to their position on the cDNA sequence.

Target gene/primer	Primer sequence 5’ -3’	Product size (bp)	Anneal temp(°C)	Start/End	Genbank accession no.
TACC3 L	TCCCTGCAGCACCGTGGGAT	341	68	364	NM001100305.1
TACC3 R	TTCGAGCTTGCTGCCCACGC			704	
AURKA L	TCGGGAGGACTTGGTTTCTT	234	65	9	DQ334808.1
AURKA R	TGTGCTTGTGAAGGAACACG			242	
TPX2 L	AACTGCGAAAGCATCCTCCA	268	65	1128	NM001098898.1
TPX2 R	TGTTTGGTTTGCAGCACAGG			1395	
GAPDH L	AGGCATCACCATCTTCCAG	326	61	179	AJ000039
GAPDH R	GGCGTGGACAGTGGTCATAA			504	
SDHA L	GCAGAACCTGATGCTTTGTG	185	64	1718	NM174178
SDHA R	CGTAGGAGAGCGTGTGCTT			1902	

### Reverse transcriptase polymerase chain reaction (RT-PCR)

RT-PCR was carried out in 200 μl tubes (Bioplastics, Landgraaf, The Netherlands) using 1 μl of cDNA as template in 25 μl PCR mixture containing final concentrations of 2 mM MgCl_2_, 200 μM of each NTP, 0.5 μM of each primer and 0.625 units Taq DNA polymerase (HotStarTaq, Qiagen) in 1xPCR buffer (Qiagen). Initial denaturation was performed at 95°C for 15 min, followed by 40 cycles of 15 sec at 94°C, 30 sec at the primer specific annealing temperature (Table 1), and 45 sec at 72°C. Final extension was performed at 72°C for 10 min. The PCR products were resolved by electrophoresis in 1% agarose gels containing ethidium bromide. A 100 base pair (bp) DNA ladder (Invitrogen) was included as a reference for fragment size.

### Quantitative RT-PCR

Quantitative RT-PCR (qRT-PCR) was performed on cDNA samples in duplicates, and singularly for the–RT blanks. Samples were quantified simultaneously in one run on a 96-well plate using a real-time PCR detection system (MyiQ Single-color Real-Time PCR Detection System; Bio-Rad Laboratories, Hercules, CA, USA). Standard curves were constructed using 10-fold serial dilutions of PCR products. The qRT-PCR reaction mixture (25 μl) contained 1 μl cDNA, 0.5 μM of each primer (Isogen Bioscience BV, Maarssen, the Netherlands) and 12.5 μl of IQ Sybr Green Supermix (Bio-Rad Laboratories). Initial denaturation took place at 95°C for 3 min, followed by 40 cycles each consisting of 95°C for 15 sec, the primer specific annealing temperature (Table 1) for 30 sec, and 72°C for 45 sec. Melting curves were plotted after the end of each PCR to verify the purity of the products. The relative starting quantity for each experimental sample was calculated based on the standard curve made for each primer pair. Data normalization was performed using *GAPDH* and *SDHA* as housekeeping genes with the same set of samples.

### Immunofluorescence

Oocytes and embryos were fixed for 30 min in 4% paraformaldehyde (PFA). After fixation, oocytes were briefly washed with 0.1% Triton X-100 and 10% FCS in PBS (PBST) and permeabilized for 30 min using 0.5% Triton X-100 in PBS; non-specific binding was then blocked by incubation for 1 h in PBST. Incubation with the primary antibody [custom made rabbit polyclonal TACC3 antibody 1:100 or anti-Aurora A, NB100-1641 1:100 (Novus Biological, Littleton CO, USA)] was performed overnight at 4°C. The oocytes and embryos were then washed three times for 15 min in PBST, followed by incubation with their respective AlexaFluor-conjugated secondary antibodies [goat anti-mouse or goat anti-rabbit IgG, 1:100 (AlexaFluor 488, Life Technologies)] for 1 h in the dark. After four washes of 20 min each, oocytes and embryos were stained with 4',6-diamidino-2-phenylindole (DAPI) for 5 min and mounted on a slide with Vectashield (Vector Laboratories, Burlingame, CA, USA). For α-tubulin staining, oocytes were first incubated for 30–60 min in microtubule stabilizing solution [19] at 37°C and then fixed in 4% PFA. After fixation, the oocytes were washed in PBS with 0.1% (v/v) Tween-20, incubated for 5 min in PBS with 2% (v/v) goat serum (G6767, Sigma, St Louis, MO, USA) followed by 60 min incubation with monoclonal anti-α-Tubulin antibody (T9026, Sigma) at 37°C. After washing, oocytes were washed and incubated with a conjugated secondary goat anti-mouse IgG (Alexa Fluor 488) for 1 h. Slides were examined by confocal laser scanning microscopy (Leica TCS SPE II, Wetzlar, Germany).

### Immunoblotting

Cells and tissues were snap-frozen and stored at -80°C until use. In order to include dividing bovine somatic cells, cumulus cells from GV-stage oocytes were isolated by vortexing, and cultured in RPM1 (Gibco, Grand Island NY, USA) supplemented with 10% FBS and 1% Pen/Strep. Samples were lysed using RIPA buffer (Pierce Biotechnology, Rockford, IL, USA) supplemented with protease/phosphatase inhibitor (Thermo, Waltham, MA, USA), based on the manufacturer’s recommendations. Proteins (20 μg per lane) were resolved on 8% and 12% SDS-PAGE gels for TACC3 and Aurora A detection, respectively, and electro-blotted to nitrocellulose membranes (Bio-Rad). Blots were blocked with 5% milk powder in TBS supplemented with 0.05% Tween20 for 1 h at room temperature and probed with either custom made anti TACC3 antibody (1/2000), anti phospho-TACC3 (ser558) antibody (1:1000, #5645, Cell Signaling, Danvers, MA, USA), anti-Aurora A, NB100-1641 (1:1000, Novus Biologicals) overnight at 4°C. After washing 3 times (15 min each) with PBST, blots were incubated for 1 h with their respective secondary antibodies (HRP-conjugated goat anti-rabbit IgG, 1:10,000; #31460; Pierce Biotechnology or HRP-conjugated goat anti mouse peroxidase, 1:5000; #sc-2005; Santa Cruz Biotechnology, Santa Cruz, CA, USA). Immunoreactivity was detected using ECL Super Signal West Dura Extended Duration Substrate (Thermo) and exposure to Kodak Bio max light film (Sigma). Blots were stripped using western blot stripping buffer (#21059, Thermo) and reprobed with anti-actin antibody (1:1000; #sc-1616; Santa Cruz Biotechnology) overnight at 4°C and subsequently progressed as described. Quantification of phosphorylated TACC3 levels was done using Quantity One software (Version 4.6.9, Bio-Rad Laboratories). Levels of actin expression were used as reference.

### Aurora A kinase inhibition

Oocytes were matured for 23 h in maturation medium in the presence of various concentrations of MLN8054 (Selleckchem, Sigma) a specific inhibitor of Aurora A kinase activity [20–22] dissolved in dimethyl sulfoxide (DMSO). As control experiments, oocytes were matured in normal maturation medium, or maturation medium containing DMSO (0.02%). Blastocyst formation rates were scored at day 8 after IVF as a percentage of blastocysts from the embryos that had been classified as cleaved on day 5.

### Injection of siRNA into oocytes

Three siRNA sequences targeting bovine *TACC3* mRNA were designed and synthesized by Dharmacon (Lafayette, CO, USA). The siRNA sequences used were: btTACC3_773: 5’-CAACAGUUCACCAAGGAAAUU-3’; btTACC3_2400: 5’- GGAAGAUCAUGGACAGCUUUU-3’; btTACC3_2607: 5’- ACGAAGAGUCACUGAAGAAUU-3’. After collection, the oocytes were denuded by vortexing for 1 min in HEPES-buffered M199 + 10% FCS. The denuded oocytes were collected in M199 + 10% FCS + 0.05IU rec hFSH (maturation medium; MM) and, until injection, placed in the incubator at 39°C in a humidified atmosphere of 5% CO_2_. Before injection, the injection mixture consisting of 2 μg/ml Tetramethylrhodamine (TRITC)-labeled 3kDa Dextran (Dextran-TRITC; Molecular Probes, Eugene, OR, USA) and the three siRNA sequences (final concentration 20 μM each) were prepared in water. Control injection consisted of injection mixture with Dextran-TRITC but without the siRNA. For injection, groups of 5 denuded oocytes were placed into drops (5 μl each) of HEPES buffered M199 + 10% FCS under oil at 37°C on an IX71 inverted microscope (Olympus, the Netherlands) equipped with a heated stage. A suction pipette was used to immobilize the oocytes and bevelled rigid borosilicated micropipettes with a 30° angle and 3.5 μm inner diameter of the tip (custom tips; Eppendorf, Hamburg, Germany) were used to perform the injections. Five μl of the injection mixture was back-loaded into the micropipettes, which were subsequently connected to a Femtojet pressure injection system (Eppendorf). Injection was performed for 0.2 seconds with pressure of 120–150 hPa. After injection, the oocytes were examined for red fluorescence by use of a TRITC filter and only the red oocytes were placed in 500μl maturation medium containing cumulus cells that had been previously removed from the oocytes. After 22-23h of maturation the oocytes were washed in PBS supplemented with 0.5% PVP (PBS-PVP) and only the oocytes that showed red fluorescence were collected. After scoring the extrusion of the first polar body by use of a stereomicroscope (50x), the oocytes were fixed in 4% (w/v) paraformaldehyde (Electron Microscopy Sciences; Hatfield, PA, USA) for 30 min at room temperature. Fixed oocytes were stored in PBS-PVP containing 1% paraformaldehyde at 4°C until immunostaining.

### Statistics

Results in bar graphs are presented as means ± standard error. Differences were tested by ANOVA with a post hoc Bonferroni test. A probability (P) below 0.05 was considered significant.

## Results

### *TACC3* is expressed in oocytes and in early cleavage stage pre-implantation embryos

**Fig 1:**
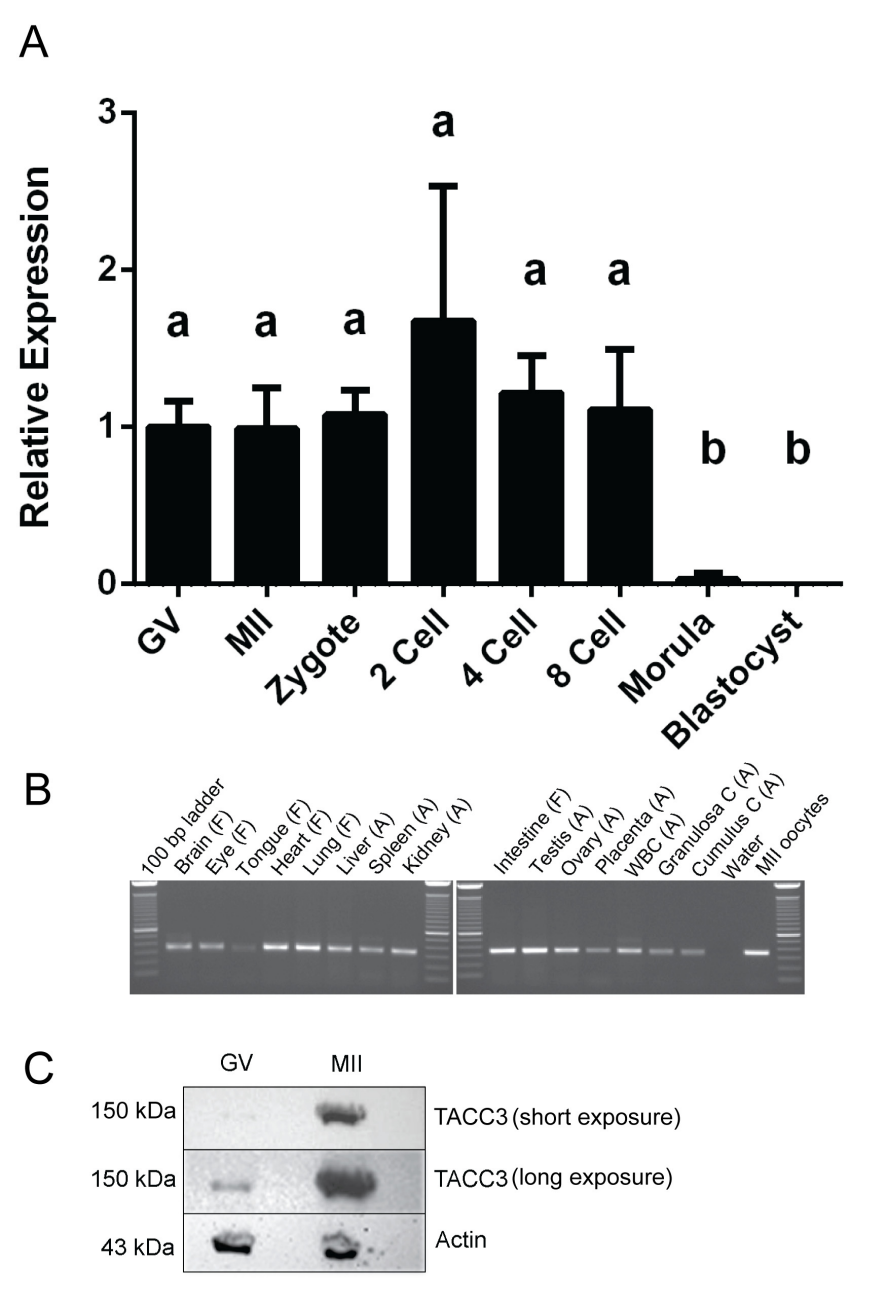
TACC3 expression in oocytes, embryos and tissues. (A) Relative expression of *TACC3* in germinal vesicle (GV) and metaphase II (MII) stage oocytes, and in pre-implantation embryos as detected by qRT-PCR. Different letters above bars (a,b) indicate values that differ significantly (p<0.05); (B) Expression of *TACC3* in bovine somatic tissues of fetal (F) and adult (A) origin as detected by conventional RT-PCR and agarose gel electrophoresis. WBC = white blood cells; (C) Immunoblot showing an increased expression of TACC3 in oocytes at the metaphase II (MII) stage compared to the germinal vesicle (GV) stage. Short and long exposure of the same blot are shown, actin is used as a loading control.

The expression of *TACC3* mRNA in bovine oocytes and *in vitro* produced pre-implantation embryos was examined using qRT-PCR. *TACC3* expression was detected at similar levels (p>0.05) throughout oocyte maturation and during the first cleavage stages (Fig 1A). However, mRNA expression was reduced significantly at the morula and blastocyst stages (Fig 1A). *TACC3* expression was also examined in bovine somatic tissue of fetal (about 8 weeks of pregnancy) and adult origin. Using conventional RT-PCR, *TACC3* mRNA expression was detected in all tissues examined (Fig 1B).

In order to examine the levels of TACC3 protein during oocyte maturation, lysates of GV and MII stage oocytes were examined by immunoblotting using a custom-made TACC3 antibody. A single protein was detected at ~150 kDa in both stages, and TACC3 expression was up-regulated at the MII stage compared to the GV stage (Fig 1C).

### Localization of TACC3 in oocytes

**Fig 2:**
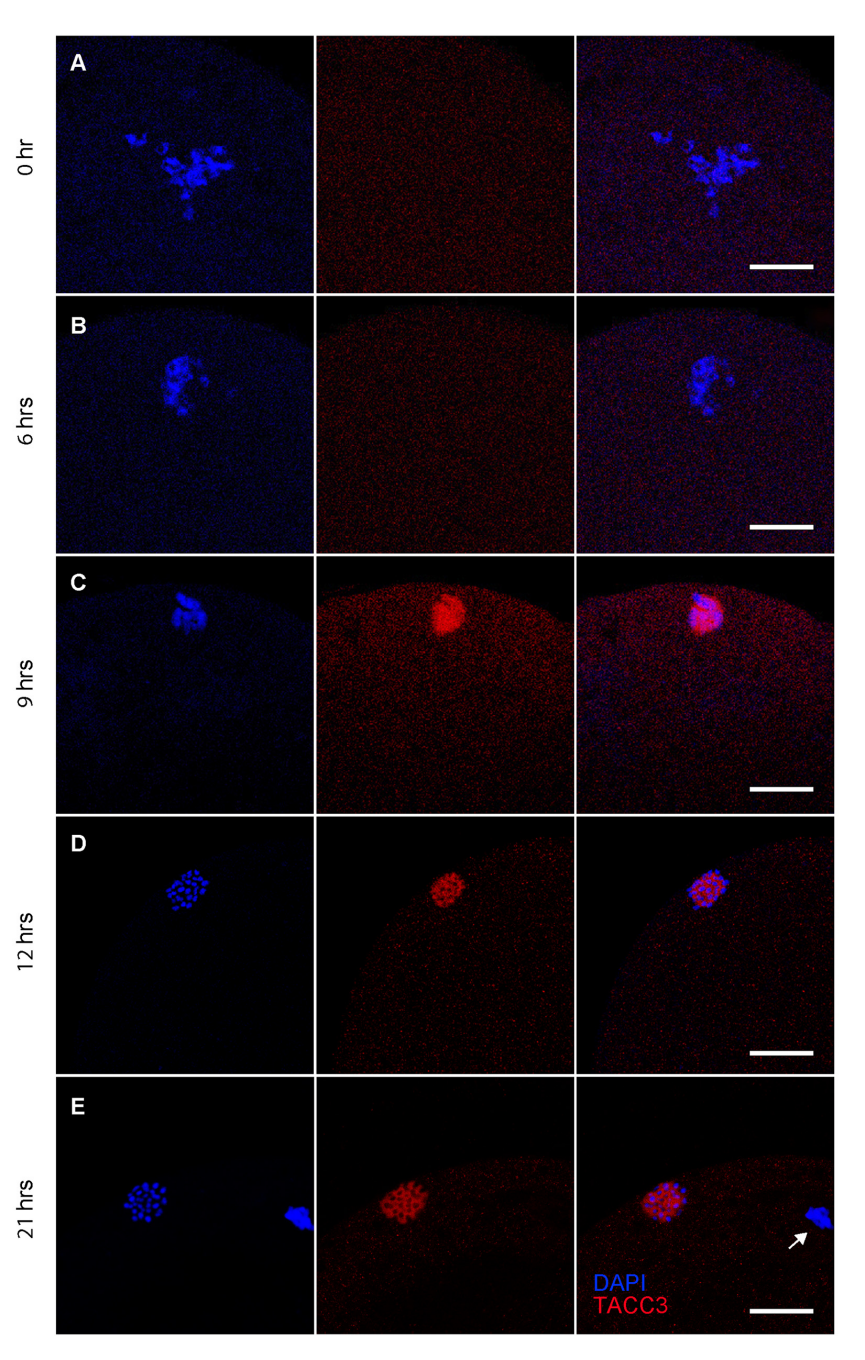
TACC3 localization in meiotic oocytes as detected by using immunofluorescence. Each row of three images are in the order; DAPI staining, TACC3 staining and overlay, and correspond to the same developmental time point. (A) 0 h, germinal vesicle stage; (B) 6 h of maturation; (C) 9 h; (D) 12 h, metaphase I stage; (E) 21 h, metaphase II stage. Arrow = first polar body. Scale bar = 20 μm.

**Fig 3:**
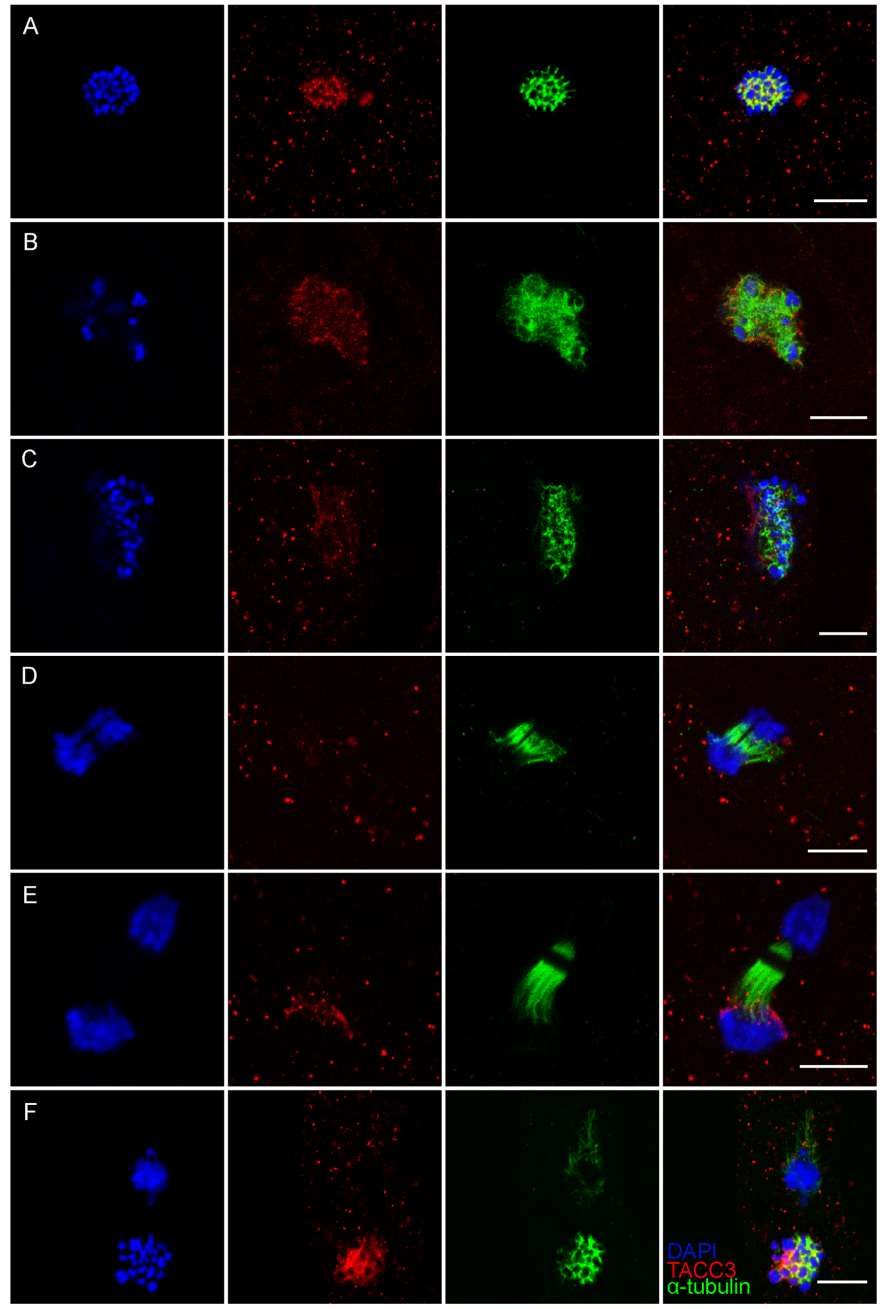
TACC3 co-localization with tubulin. TACC3 co-localizes with tubulin in the spindle at metaphase I, is not expressed at anaphase and reappearance at telophase. TACC3 is not present in the polar body. (A-C) metaphase I stage, (D) anaphase stage, (E) telophase stage and (F) metaphase II stage; blue = DAPI/DNA. Red = TACC3 and green = α-tubulin; Scale bar = 10 μM.

In order to determine the subcellular localization of TACC3, whole mount immunofluorescence was performed on *in vitro* matured oocytes. Although low levels had been detected by immunoblotting, TACC3 expression could not be detected in GV stage oocytes using immunofluorescence (Fig 2A and 2B). Instead, TACC3 immunofluoresence was first detected 9 hours after the onset of maturation culture, situated around the genomic DNA that was exhibiting the first signs of condensation shortly prior to formation of the first metaphase spindle (Fig 2C). After 12 h of maturation, and after the first metaphase spindle had formed, TACC3 was visualized tightly surrounding the paired chromosomes (Fig 2D) and apparently co-localized with the tubulin of the spindle (Fig 3A). After 15 and 16.5 h of IVM, when the homologous chromosomes were still aligned, TACC3 protein could be seen at both sides of the spindle (data not shown). As the oocyte entered telophase of the first meiotic division, TACC3 was no longer detectable although a clear spindle was visible after staining for α-tubulin (Fig 3B and 3C). After 18 h of maturation, shortly before the second metaphase spindle was complete, TACC3 expression re-appeared and was now localized around the chromosomes in the spindle but not around the DNA that was condensing prior to extrusion as the first polar body (Fig 3E). When the metaphase II spindle was complete at 21 h of IVM, TACC3 was localized around the chromosomes within the spindle (Fig 2E), and co-localized with tubulin (Fig 3F) similar to the pattern observed during the first metaphase stage; however, almost no TACC3 was visible around the DNA in the first polar body (Fig 2E and 3F).

Following fertilization *in vitro*, the reactivation and completion of the second meiotic division was analyzed in presumptive zygotes. At 4 h post fertilization, TACC3 was localized around the sister chromatids that were still aligned at the equator of the meiotic spindle. As the sister chromatids started to separate, TACC3 expression decreased quickly and was last detected around the chromosomes after the pronuclei had been formed but before they had fused (data not shown).

### Expression of Aurora A during oocyte maturation

**Fig 4:**
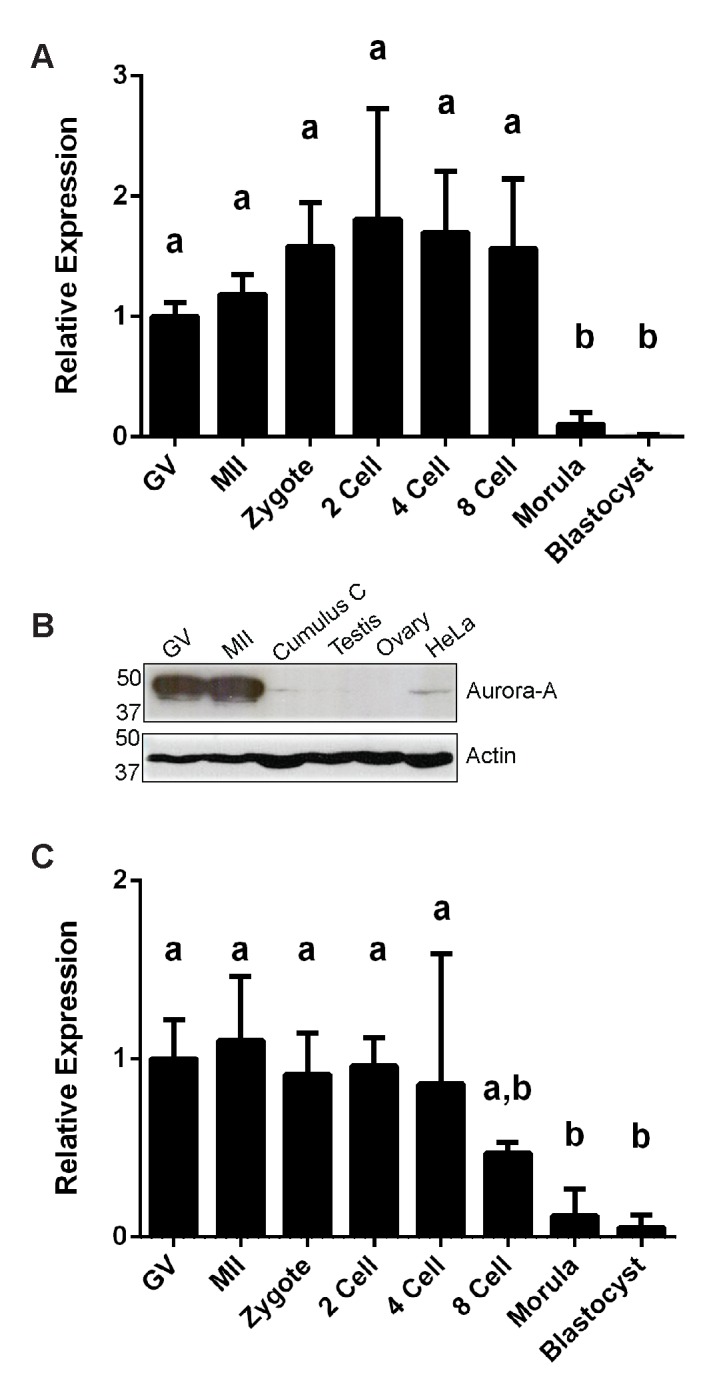
Aurora A and TPX2 expression. (A) Relative expression of *AURKA* by qRT-PCR in oocytes and pre-implantation embryos; (B) Western blot for Aurora A expression, actin is used as loading control; (C) Relative expression of *TPX2* in oocytes and pre-implantation embryos. GV, MI, MII, 2 Cell, 4 Cell, 8 Cell, correspond to germinal vesicle, metaphase I, metaphase II, 2-cell embryo, 4-cell embryo, and 8-cell embryo, respectively. Different letters above bars (a,b) indicate values that differ significantly (p<0.05).

Since it has been reported that phosphorylation by Aurora A is required for TACC3 to localize at the mitotic spindle [7], expression of the gene coding for Aurora A, *AURKA*, was examined. *AURKA* was expressed at similar levels in oocytes and early developing embryos; however, expression levels dropped significantly (p<0.05) from the morula stage onwards (Fig 4A). Immunoblotting using an antibody directed against human Aurora A confirmed the expression of Aurora A in bovine GV and MII oocytes. By contrast, very little expression was detected in cumulus cells or whole testis lysate, and no expression was detected in whole ovarian lysate. Mitotic HeLa cells were used as a positive control (Fig 4B).

Since it has been described that Aurora A is activated by TPX2 during mitosis [11,12] we also examined *TPX2* mRNA expression in oocytes and early embryos. *TPX2* expression showed a similar pattern to *TACC3* and *AURKA*, i.e. an obvious decrease at the morula and blastocyst stages (Fig 4C).

**Fig 5:**
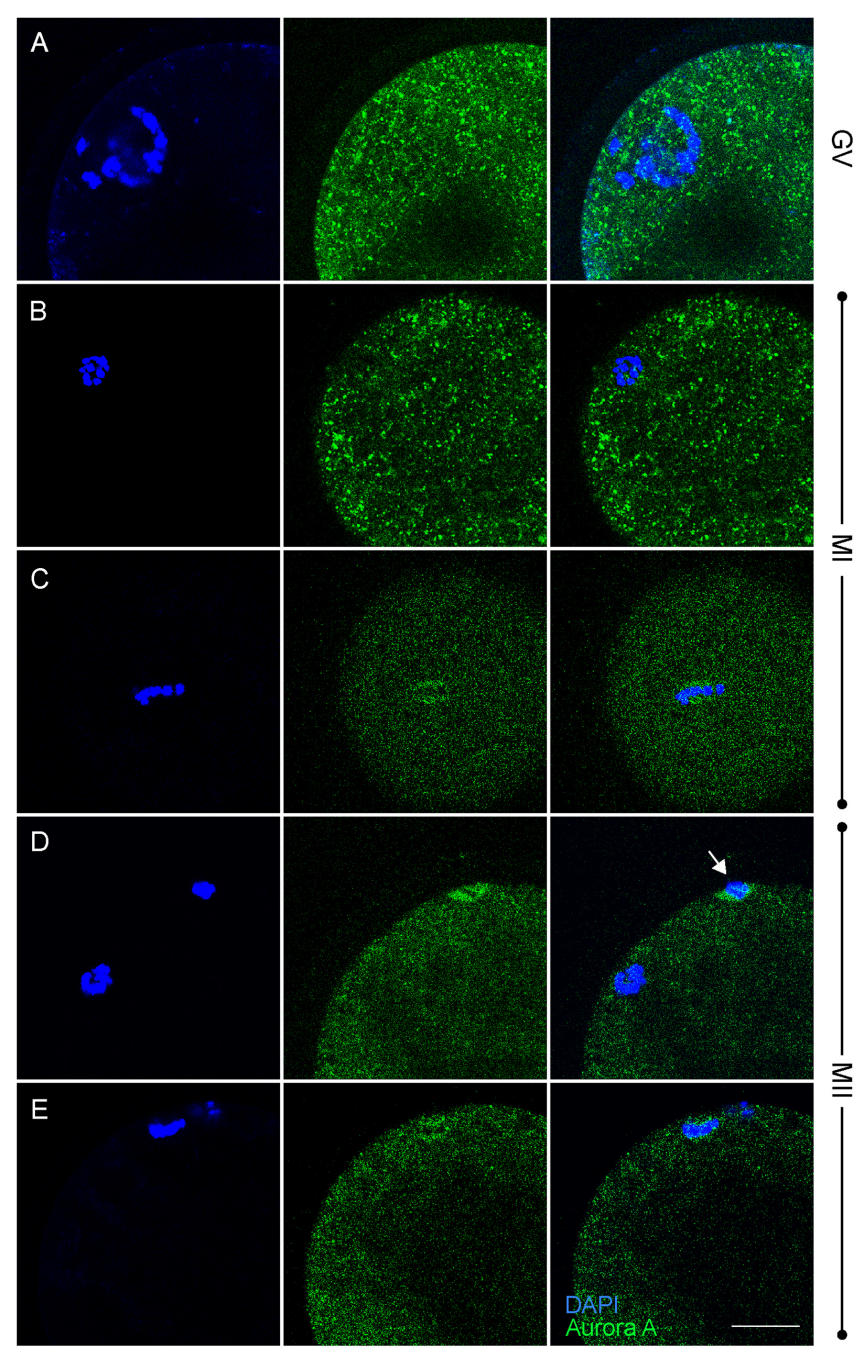
Aurora A localization in meiotic oocytes. Punctuated expression of Aurora A in GV until MII oocytes, infrequent expression around chromosomes at MI and MII stages and aggregation at polar body. Each row of three images is in the order DAPI staining, Aurora A staining and overlay; correspond to the same developmental time point. (A) Germinal vesicle oocyte; (B, C) 12 h of IVM; metaphase I stage; (D, E), 23 h of IVM; metaphase II stage; Scale bar = 25 μM. Arrow = first polar body.

In order to monitor the spatio-temporal localization of Aurora A, whole mount immunofluorescence was performed on oocytes at different stages of *in vitro* maturation. Punctate expression of Aurora A was detected in oocytes of all investigated stages from GV until MII (Fig 5). In a small percentage (<10%) of the metaphase I (Fig 5C) and metaphase II (Fig 5E) oocytes Aurora A was concentrated around the chromosomes, suggesting a very transient expression at the spindle. Unexpectedly, Aurora A was also detected within the extruded first polar body (Fig 5D).

### Indirect inhibition of TACC3 using MLN8054 disrupts meiotic spindle formation in a dose-dependent manner

**Fig 6:**
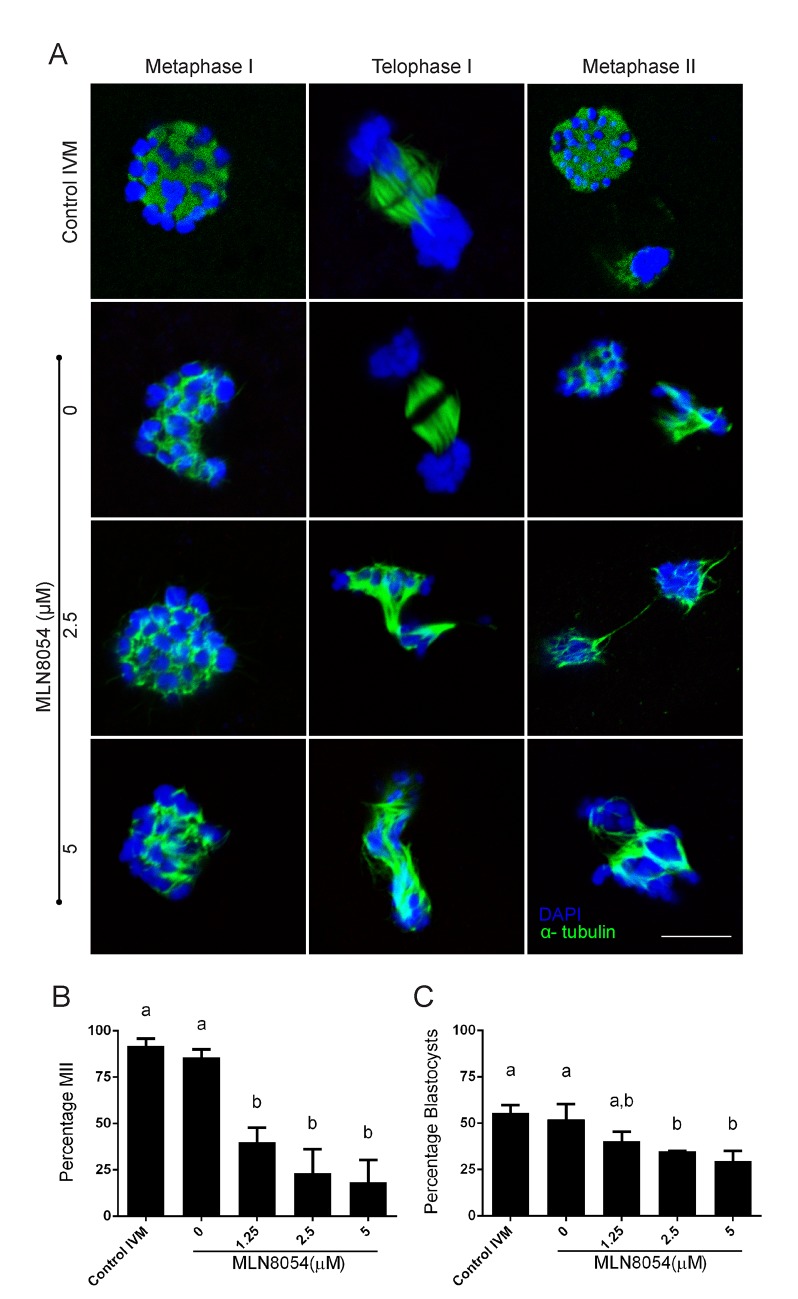
Inhibition of Aurora A activity disrupts meiotic spindle formation. (A) Abnormal spindles and misaligned chromosomes after inhibition of Aurora A using MLN8054. Microtubules (immunostaining) are in green, blue represents DNA (DAPI). Scale bar = 10 μm; Effect of various concentrations of MLN8054 on the percentages of (B) oocytes progressing through meiosis to reach MII and (C) blastocyst formation from MII oocytes.

**Fig 7:**
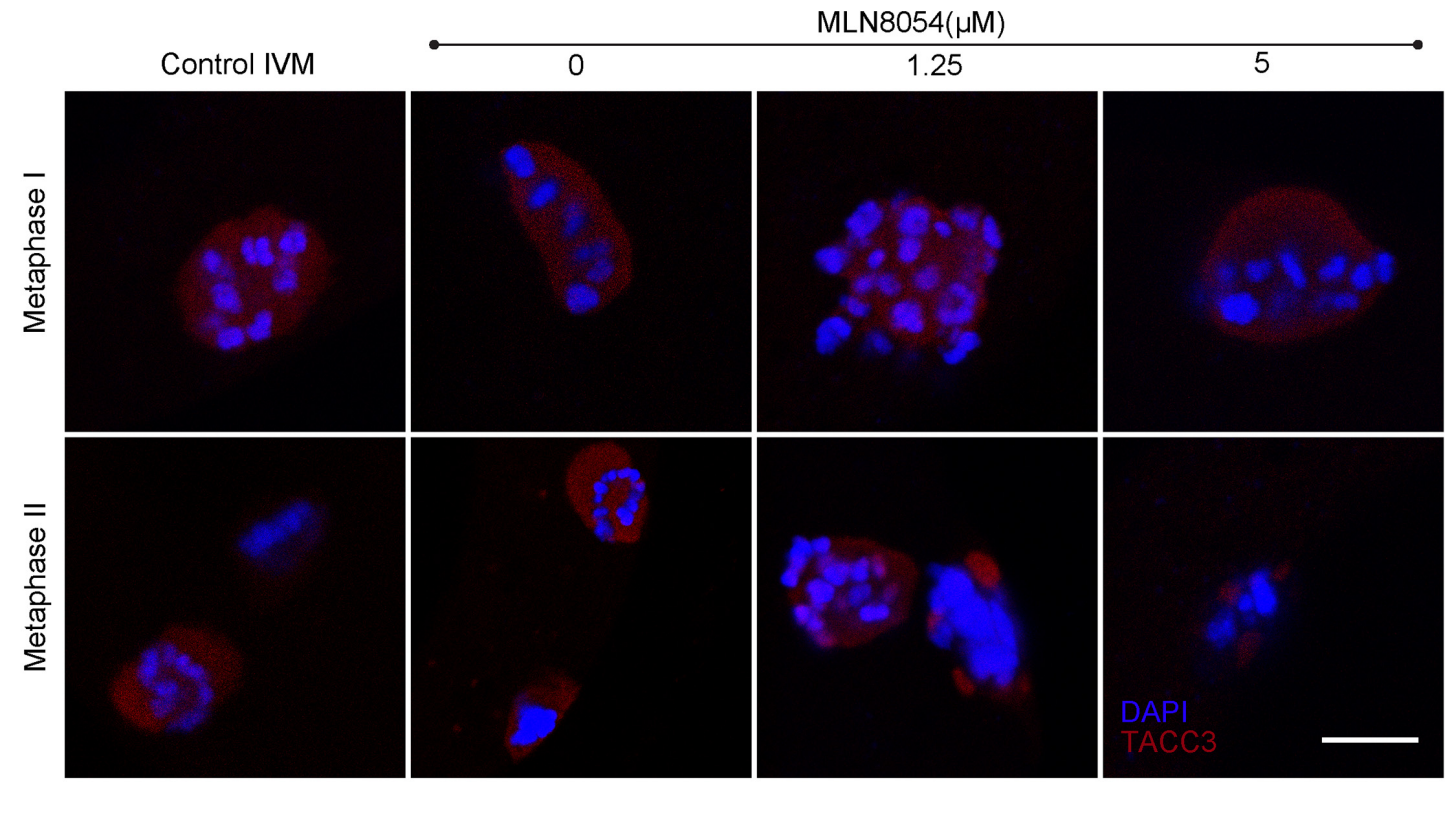
TACC3 expression after exposure of bovine oocytes to MLN8054 during maturation *in vitro*. Shown are cells at metaphase I (upper row) and metaphase II (bottom row) cultured at the indicated concentrations of MLN8053. TACC3 expression in red is detected with immunostaining; blue represents DNA (DAPI). Scale bar = 10μM.

It has been suggested that localization of TACC3 to the mitotic spindle is dependent on phosphorylation by Aurora A [7–9]. Indeed, inhibition of Aurora A in human tumour cells using MLN8054 [21,22] leads to aneuploidy and chromosomal defects [21]. When bovine oocytes were matured in the presence of MLN8054 the levels of phosphorylated TACC3 (pTACC3) decreased to 77% of the control levels (77 ±12; n = 3). Oocytes with decreased pTACC3 levels after MLN8054 exposure exhibited abnormal meiotic spindles and misaligned chromosomes (Fig 6A). Note that the control spindles at metaphases appear circular as they are imaged perpendicular to the orientation of the spindle. The chromosomes are however properly aligned as can be seen when the spindle is imaged from a different angle (S1 avi). Abnormal spindle formation was most prominent at the metaphase II stage, although most oocytes exposed to the highest concentrations of MLN8054 did not even progress to this stage. Indeed, the percentage of oocytes that reached metaphase II as determined by the presence of a polar body was reduced in a MLN8054 concentration dependent manner (Fig 6B), with the majority of oocytes arresting during the first meiotic division. Although oocytes that had reached the MII stage in the presence of MLN8054 appeared morphologically normal, a smaller percentage developed to blastocysts after fertilization as compared to non-exposed control oocytes (Fig 6C). On the other hand, no significant difference in TACC3 expression was observed at the metaphase I stage in oocytes that had been matured in the presence of MLN8054 (Fig 7). In metaphase II oocytes subjected to Aurora A inhibition, however, TACC3 was expressed in clusters around the DNA rather than being evenly distributed over the meiotic spindle (Fig 7). Inhibition of Aurora A by MLN8054 during oocyte meiosis did not noticeably affect the expression of *TACC3*, *AURKA* or *TPX2* mRNA (p>0.05) compared to the levels in control oocytes (S1 Fig.).

### Inhibition of *TACC3* expression by siRNA injection

**Fig 8:**
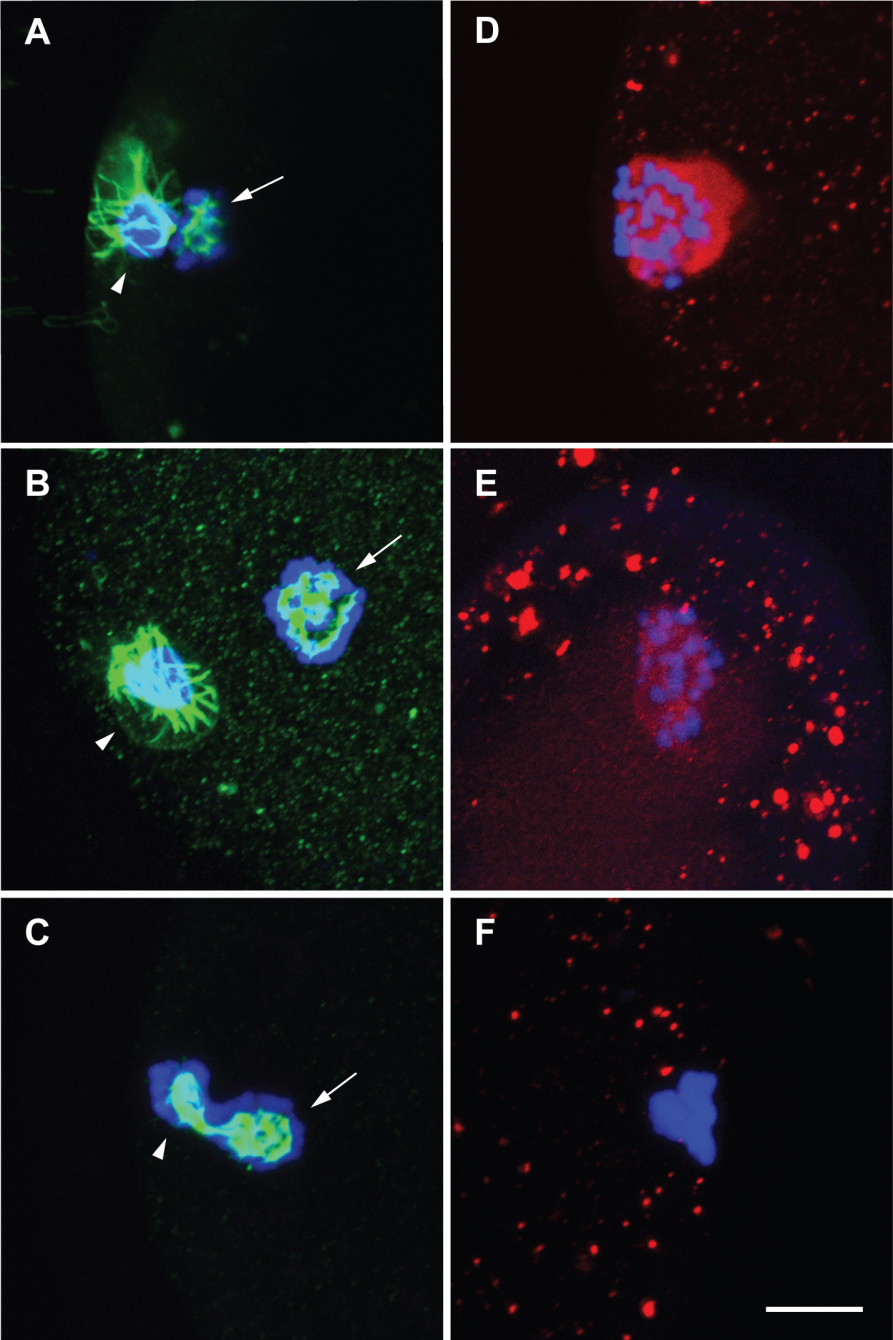
Tubulin and TACC3 expression in oocytes after siRNA injection against TACC3. Oocytes were injected at the GV stage and thereafter matured for cultured for 23 h. The cells are immunostained for tubulin (green; A,B,C) and TACC3 (red; D,E,F) and stained with DAPI (blue) for DNA. (A,D) Non-injected control; (B,E) TRITC-injected; (C,F) siRNA injected. Metaphase spindle (arrow) and polar body (arrowhead) are indicated. Scale bar = 10μM.

The expression of TACC3 was also directly knocked-down via injection of siRNA directed against TACC3 into GV stage oocytes. After injection the oocytes were cultured for 23 h after which they were fixed and immunostained for TACC3 and tubulin expression. After injection of TACC3-siRNA the oocytes failed to mature properly. Fewer (22.7%, n = 22) oocytes succeeded in extrusion of the first polar body after TACC3-siRNA injection compared with TRITC-injected (42.9%, n = 28) and non-injected (68.4%, n = 117) oocytes. Indeed the expression of TACC3 was severely reduced after siRNA injection, only in 7% of the siRNA injected oocytes could TACC3 be detected (1 out of 14 injected oocytes, and this oocyte had not extruded a polar body). Of the control oocytes 50% (8 from 16) from the TRITC-Dextran injected oocytes and 90% (9 out of 10) non-injected oocytes showed TACC3 expression. All oocytes injected with si-TACC3 had abnormal spindles with compact chromatin (Fig 8 and S2 avi). In contrast all the oocytes in the non-injected group and 83% of TRITC injected group had normal spindles (Fig 8). These results corroborate with the Aurora A kinase inhibition suggesting that TACC3 expression is important for proper meiotic spindle formation.

## Discussion

Mammalian oocyte maturation is a complex process the regulation of which is incompletely understood. Clearly, correct segregation of the chromosomes during the two meiotic divisions is essential for formation of a normal embryo. In addition, the correct cytoplasmic structures need to be retained in the oocyte while half of the chromosomes are eliminated via the first polar body. Formation of a normal functioning spindle is essential for these processes.

During mitotic cell division, it has been shown that Aurora A-activated TACC proteins stabilize the centrosomal microtubules [9,21]. We detected *TACC3* mRNA expression in maturing bovine oocytes and early cleavage stage embryos up to the 8 cell stage, after which expression decreased significantly to reach very low levels in morulae and blastocysts. In cattle, the embryonic genome is activated at around the 8-cell stage [23]. Activation of the embryonic genome is accompanied by the degradation of maternal mRNA, which could at least in part explain the dramatic drop in expression in later stage embryos. However, *TACC3* mRNA expression was also detected in all somatic tissues examined, presumably reflecting the importance of TACC3 during mitotic cell division. Interestingly, in adult human tissues *TACC3* expression was highest in testis, whereas it was not detected in skeletal muscle, heart or brain and was present at only very low levels in the ovaries [24]. Within human ovaries, immunostaining revealed TACC3 protein expression in the cytoplasm of oocytes in developing follicles, although expression in post GVBD human oocytes has not been reported [24]. This suggests that, in somatic tissues, levels of *TACC3* expression are highest in cells with a high mitotic index; however, we did not further examine expression levels in bovine somatic tissues.

In this study we show that TACC3 exhibits a dynamic expression pattern in bovine oocytes following resumption of meiosis and GVBD. At the metaphase I and II stages, TACC3 protein was located close to the chromosomes where it appeared to co-localize with the microtubules of the meiotic spindle. TACC3 expression then disappeared during anaphase I only to reappear during the telophase of the first division close to the set of homologous chromosomes that would align along the second metaphase plate, but not associating with the set of chromosomes that would form the polar body. During both metaphases, TACC3 co-localized with α-tubulin, whereas during anaphase and telophase TACC3 did not appear to associate with α-tubulin, as determined by immunofluorescence. The disappearance of TACC3 during anaphase and reappearance during telophase indicates a rapid turnover of the protein. Whether it is degraded and newly synthesized or dispersed throughout the cytoplasm and subsequently reaggregated at the spindle is not known. Based on the high levels of *TACC3* mRNA at the MII stage, however, synthesis of new proteins is not unlikely.

TACC3 has been shown to be phosphorylated by Aurora A [7–9]. In bovine oocytes, Aurora A expression was detected at the metaphase I and II spindles, similar to what has been described in mitotic cells [24], but only in a small percentage of oocytes suggesting a transient expression of Aurora A and a transient interaction of Aurora A with TACC3. This expression of Aurora A is quite different to that described in mouse oocytes, where Aurora A was concentrated within the germinal vesicle and later became associated with the spindles [15]. These results indicate that, in bovine oocytes, TACC3 phosphorylation by Aurora A is not essential for TACC3 to localize at the meiotic spindle. Indeed, this conclusion was supported by the finding that inhibition of the kinase activity of Aurora A using MLN8054 did not prevent TACC3 from accumulating at the MI or MII spindle, even though fewer oocytes subsequently progressed to the MII stage. In addition, following Aurora A kinase inhibition TACC3 appeared to be expressed in clusters around the spindles, and the chromosomes failed to align. In order to determine whether inhibition of Aurora A kinase activity indeed led to decreased levels of pTACC3, we cultured bovine oocytes in the presence of MLN8054 and quantified pTACC3 levels by immunoblotting using an antibody that recognizes human TACC3 phosphorylated at serine 558. The corresponding serine in bovine TACC3 is serine 499 and was indeed recognized by the antibody. Quantification of the levels of pTACC3 revealed that even in the presence of the Aurora A inhibitor, the levels of TACC3 phosphorylated on ser499 were substantial, suggesting that either pTACC3 is rather stable or that TACC3 in bovine oocytes can also be phosphorylated on ser499 by other kinases. Given the downregulation of TACC3 levels in oocytes between MI and MII stages it seems unlikely that pTACC3 is stable but instead suggests phosphorylation by other kinases. When expression of TACC3 was downregulated, irrespective of the phosphorylation state, by siRNA injection into oocytes, the meiotic spindles showed abnormalities similar to those observed after inhibition of Aurora A kinase activity. Normal meiosis and extrusion of a first polar body was severely compromised after TACC3 knockdown, as also observed after Aurora A kinase inhibition, although chromosome condensation and clustering took place. Taken together, these results indicate that during meiosis in oocytes TACC3 is not essential for the formation of the spindle but is important for the stability of that spindle once formed particularly during metaphase of both divisions.

It has previously been reported that Aurora A-activated TACC3 can form a complex with both colonic and hepatic tumour-overexpressed gene (chTOG) and clathrin to stabilize microtubules within the mitotic spindle [9,25]. Interestingly, the spindle abnormalities observed after indirect TACC3 inhibition and siRNA-directed TACC3 knockdown are similar to those described after inhibition of clathrin in porcine oocytes [26]. Whether TACC3 phosphorylation is however essential for TACC3 to form a complex with clathrin during meiosis is not known.

Unexpectedly, Aurora A was present in the first polar body of all MII oocytes examined. In mouse oocytes, Aurora A was not detected in the first polar body but instead remained associated with the second meiotic spindle [15]. It has previously been reported that in polar bodies of bovine oocytes Aurora A localizes at the mid-body, suggesting a function in cytokinesis [27]. Moreover, both inactivation and over-activation of Aurora A result in aberrant cytokinesis, suggesting that timely inactivation of Aurora A and dephosphorylating of its targets may be essential for the completion of cell division. In this study, we detected Aurora A expression throughout the polar body. Possibly Aurora A and its protein partners accumulate at the cleavage furrow required for polar body removal from the oocyte. However, having played a role in this modified cell division, they could be removed from the oocyte, and thereby essentially inactivated, by partitioning into the polar body. Clathrin expression has also been described in the polar body of porcine oocytes [26]. On the other hand, we did not detect TACC3 in the polar body which presumably excludes formation or maintenance of a TACC3-chTOG-clathrin complex in these structures. Surprisingly, while investigating binding partners for TACC3 proteins by immunoprecipitation followed by mass spectrometry, we detected ch-TOG but neither clathrin nor Aurora A (data not shown). While it is not clear why Aurora A was not detected as a co-precipitate it could be due to the transient nature of the interaction between TACC3 and Aurora A. Future work will have to determine which other proteins interact with TACC3 during meiosis and whether the function of TACC3 in oocyte meiosis is conserved among mammals.

## Supporting Information

S1 avi 3D movie of the spindle of a control oocyte at the metaphase II stage. Both the spindle and the polar body are visible. Tubulin expression in green; blue represents DNA (DAPI).
(AVI)

S2 avi 3D movie of the spindle of a TACC3-siRNA injected oocyte at the metaphase II stage. Tubulin expression in green; blue represents DNA (DAPI). This is the same oocyte as shown in Fig 8C.
(AVI)

S1 Fig Effect of MLN8054 on gene expressions . Relative expression of *TACC3* (A), *AURKA* (B) and *TXP3* (C) in oocytes cultured with various concentrations of MLN8054 for 12 and 23 h.
(TIF)
