# Supplementary figures and images for: TACC3 Is Important for Correct Progression of Meiosis in Bovine Oocytes
Source: PLoS One. 2015 Jul 13;10(7):e0132591. doi: 10.1371/journal.pone.0132591 (PMC4500572; doi:10.1371/journal.pone.0132591)

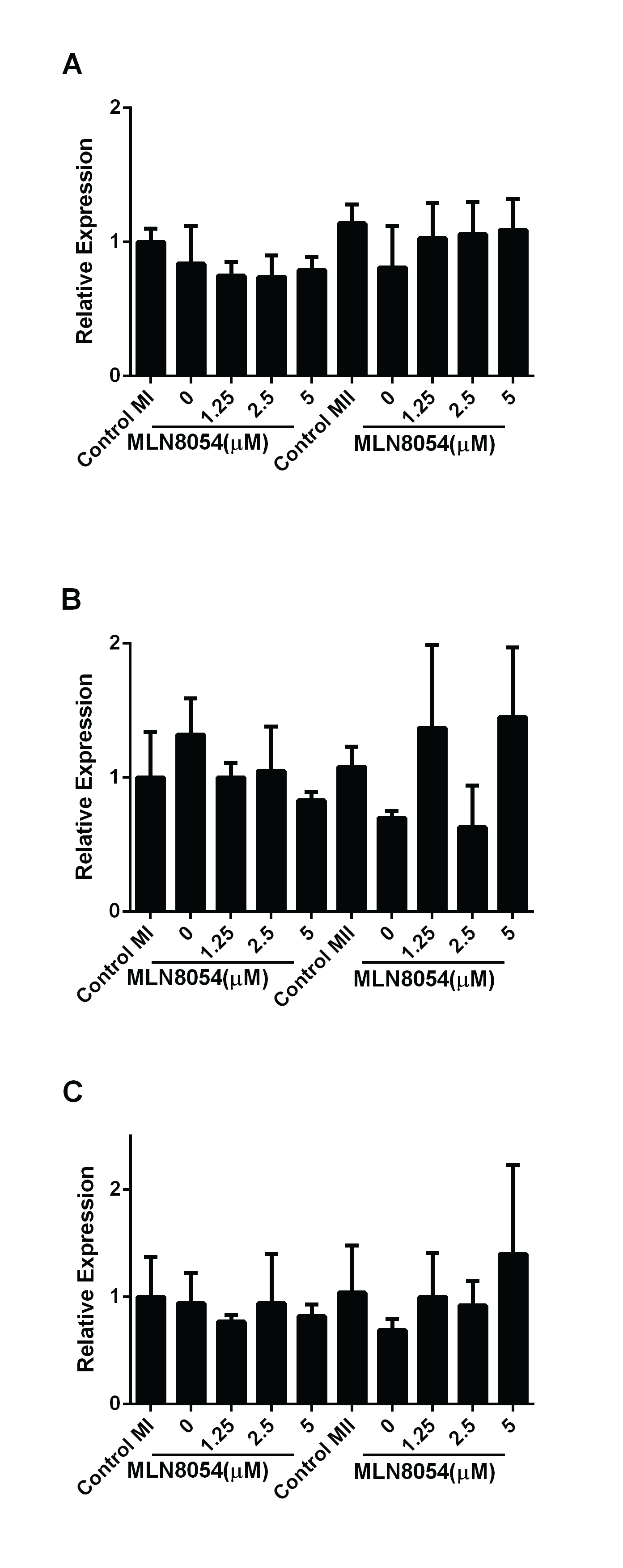

Supplement: S1 Fig — . Relative expression of TACC3 (A), AURKA (B) and TXP3 (C) in oocytes cultured with various concentrations of MLN8054 for 12 and 23 h. (TIF) [file pone.0132591.s003.tif]
